# An audit of the adequacy of contrast enhancement in CT pulmonary angiograms in a South African tertiary academic hospital setting

**DOI:** 10.4102/sajr.v26i1.2350

**Published:** 2022-03-24

**Authors:** Derik J. Basson, Halvani Moodley

**Affiliations:** 1 Department of Diagnostic Radiology, Faculty of Health Sciences, University of the Witwatersrand, Johannesburg, South Africa; 2 Department of Radiology, Charlotte Maxeke Johannesburg Academic Hospital, Johannesburg, South Africa; 3 Faculty of Health Sciences, University of the Witwatersrand, Johannesburg, South Africa

**Keywords:** contrast enhancement, audit, CT pulmonary angiogram, pulmonary embolism, flow rate

## Abstract

**Background:**

Undiagnosed pulmonary embolism carries high mortality and morbidity. Computed tomography pulmonary angiogram (CTPA) is the diagnostic method of choice for accurate diagnosis. Inadequate contrast opacification is the second most common cause of indeterminate CTPAs.

**Objectives:**

Audit the adequacy of CTPA contrast enhancement and determine whether inadequate enhancement is affected by the size and site of the intravenous cannula, flow rate, contrast volume, contrast leakage and day shift versus after hours services.

**Method:**

Retrospective and prospective audits of the adequacy of contrast enhancement of CTPAs at the Charlotte Maxeke Johannesburg Academic Hospital were conducted using the Royal College of Radiologists guidelines (≤ 11% of studies with < 210 HU). Protocol variables were collected prospectively from questionnaires completed by radiographers performing the CTPAs. Adequate versus inadequate groups were analysed.

**Results:**

A total of 63 (retrospective) and 130 (prospective) patients were included with inadequate contrast enhancement rates of 19% (12/63) and 20.8% (27/130), respectively. The majority of CTPAs were performed during the day 56.2% (73/130) with a 20G cannula 66.2% (86/130) in the forearm 33.8% (44/130) injecting 100 mL – 120 mL contrast 43.1% (56/130) at 3 mL/s 63.1% (82/130). The median flow rate (3 mL/s) and contrast volume (80 mL) were identical in both adequate and inadequate groups, while the remaining variables showed no statistical difference.

**Conclusion:**

The rate of inadequately enhanced CTPAs in this study was high. The protocol variables did not have a significant influence on the rate of inadequate enhancement. Further research, particularly using flow rates > 4 mL/s, is required for protocol optimisation.

## Introduction

Accurate diagnosis of pulmonary embolism (PE) is imperative as untreated or undiagnosed PE carries a high mortality and morbidity.^1^ Computed tomography pulmonary angiogram (CTPA) is the method of choice in the diagnosis of PE;^2,3^ however, indeterminate studies have been estimated at 6.6%.^4^ Inadequate contrast opacification has been cited as the second most common cause of indeterminate CTPA studies, the first being motion artefact, while less common causes include beam hardening artefact related to obesity and streak artefact.^4,5^ The exact prevalence of PE in South Africa is not known; however, in 2016, 2124 deaths due to pulmonary vascular disease were reported, which included PE.^6^

The Royal College of Radiologists (RCR) recommend audits of CTPAs in order to limit indeterminate CTPAs due to inadequate enhancement. The RCR guideline (2013) advocates that a maximum of 11.0% of CTPAs can have inadequate contrast enhancement (< 210 Hounsfield units [HU]).^7^ Several international audits have been performed, reporting a percentage of CTPA studies with inadequate contrast opacification varying between 1.5% and 18.0%.^8,9,10,11^ Some had decreased inadequate rates following protocol changes, which were prompted by an audit.^9,11^

To the best of our knowledge, no such audit has been performed in South Africa. This audit primarily assessed the adequacy of contrast enhancement of CTPA examinations at a South African tertiary radiology department and secondarily assessed the influence of certain technical factors.

## Materials and methods

A single-centre retrospective and prospective audits were performed at a large tertiary hospital in South Africa, which included routine in- and outpatient CTPA referrals from this hospital as well as from a network of referral hospitals and clinics.

The retrospective audit was conducted using all consecutive CTPA studies for suspected PE in adult patients (18 years or older) from 01 December 2019 to 31 December 2019, while the prospective audit included all consecutive CTPA studies from 01 January 2020 to 31 March 2020. Informed consent was obtained from the patients for the prospective audit. Additionally, the prospective audit incorporated a questionnaire that was completed by the attending radiographer acquiring the scan, which included technical factors such as intravenous (IV) cannula size, location of the IV cannula, flow rate of the injector, volume of contrast, presence of contrast leakage and the time at which the scan was acquired. Data from the radiographer checklists were captured.

CT pulmonary angiograms were performed on either a Phillips 64 slice Brilliance, 128 slice Ingenuity or Siemens 64 slice Somatom CT scanner. Examinations were performed with the patient in the supine position with arms placed above the head and scans acquired in a cranio-caudal direction, from the lung apices to the mid-low liver. Patients were instructed to inspire and hold their breath, after which scanning was performed at full inspiration. A dose of 80 mL – 100 mL 350 mg iodine/mL of non-ionic iodinated intravenous contrast material (Omnipaque) was administered through the IV cannula, via a Guerbet Optivantage pump injector at a rate determined by the radiographer (preferably > 3 mL/s), depending on the IV-line size and position. Bolus tracking was used with a region of interest (ROI) centred within the main pulmonary artery (MPA), at the level of its bifurcation, with scanning to be initiated after a 6.2 s delay once a level of 110 HU was reached within the ROI.

**FIGURE 1:**
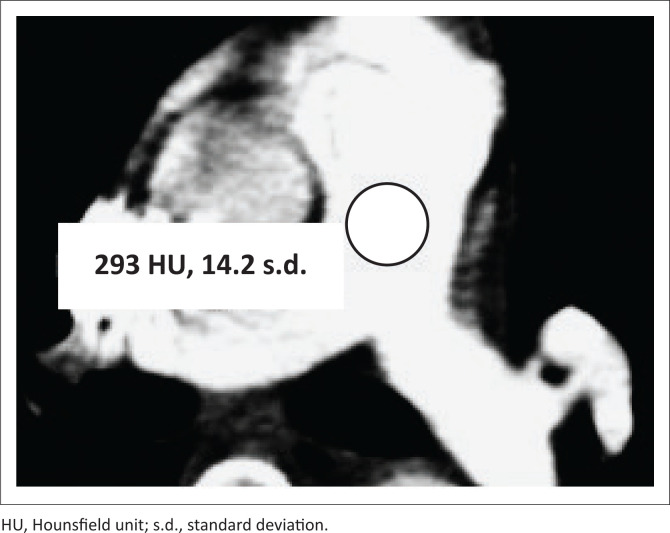
Contrast enhancement: Hounsfield unit measurement in the main pulmonary artery: 293 HU (adequate enhancement).

All images were viewed on a Philips Enterprise PACS workstation. The contrast enhancement of every CTPA was assessed by placing a circular ROI within the MPA at its largest axial diameter on each study at a slice thickness of 1 mm. The ROI diameter was 50% of the vessel diameter. The HU obtained at this level was recorded as an average of three measurements (Figure 1). Hounsfield unit readings in the MPA were captured, and the HU was further categorised as adequate (≥ 210 HU) or inadequate (< 210 HU).

### Statistical analysis

Descriptive statistics (frequencies and percentages) were calculated for categorical data, and medians and percentiles for numerical data. The Shapiro-Wilk test was used to investigate whether numerical variables followed a normal distribution. The Chi-Squared or Fisher’s exact tests were used to compare frequencies of adequate versus inadequate HU values. The Mann–Whitney *U*-test was used to compare median values for the two independent groups: adequate HU versus inadequate HU. A significance level (α) of *p* < 0.05 was used. All data analysis was performed using SAS version 9.2.

### Ethical considerations

Ethics approval was provided by the Committee of Human Research of the Faculty of Health Sciences, University of the Witwatersrand (ethics clearance number M190454). Informed consent was obtained from the patients for the prospective audit. The identifying data of patients were anonymised and stored on a password-protected computer.

## Results

A total of 63 (retrospective) and 149 patients (prospective) were included. Nineteen CTPAs from the prospective audit were excluded due to incomplete informed consent. Inadequate contrast enhancement was identified in 19% (12/63) of the retrospective and 20.8% (27/130) of the prospective cases.

The majority of CTPAs were performed with a 20 G IV cannula (86/130, 66.2%), mostly in the forearm (44/130, 33.8%), followed by the hand and antecubital fossa. The IV cannula sizes were not significantly different between the adequate and inadequate contrast enhancement groups (*p* = 0.20). Similarly, the IV cannula sites were not significantly different between the two groups (*p* = 0.65). (Table 1).

**TABLE 1:** CTPA technical factors.

Technical factors	Frequency (*n*=130)				*p*-value
	Adequate		Inadequate		
	*n*	%	*n*	%	
**IV cannula size**					0.20
Central venous catheter	7	5.4	0	0.0	
22 G	0	0.0	1	0.8	
20 G	67	51.5	19	14.6	
18 G	29	22.3	7	5.4	
**IV cannula site**					0.65
Subclavian vein	3	2.3	0	0.0	
Internal jugular vein	3	2.3	0	0.0	
Femoral vein	1	0.8	0	0.0	
Antecubital fossa	29	22.3	8	6.1	
Forearm	37	28.5	7	5.4	
Hand	30	23.1	12	9.2	
**Flow rate**					0.59
> 4ml/s	6	4.6	0	0.0	
< 4ml/s	97	74.6	27	20.8	
**Time**					0.21
Day shift	55	42.3	18	13.8	
Afterhours	48	36.9	9	7.0	
**Contrast leakage**					0.60
No	99	76.2	25	19.2	
Yes	4	3.1	2	1.5	

IV, intravenous.

**FIGURE 2:**
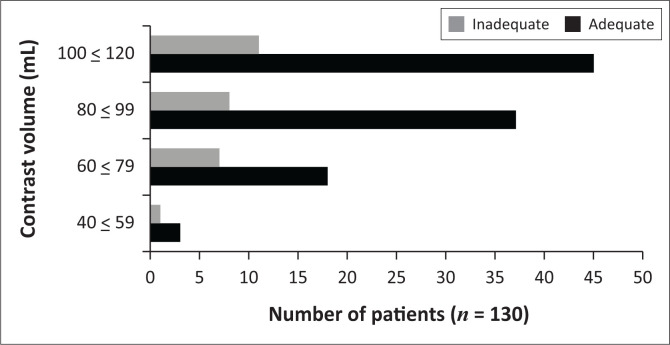
Frequency of adequate and inadequate contrast-enhanced CTPAs in the prospective group of patients for different contrast volumes.

**FIGURE 3:**
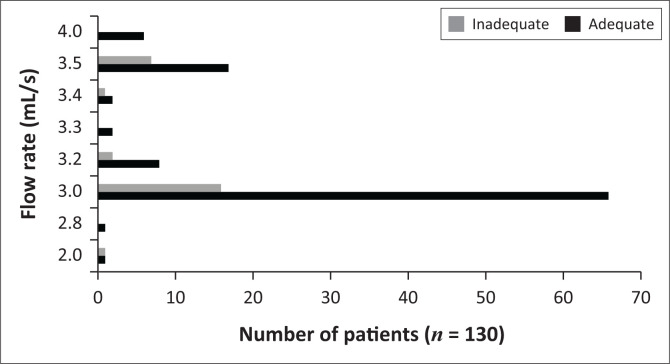
Frequency of adequate and inadequate contrast-enhanced CTPAs in the prospective group of patients for different flow rates.

The majority of CTPAs were performed with 100 mL – 120 mL of contrast (56/130, 43.1%) (Figure 2) at 3 mL/s (82/130, 63.1%) (Figure 3). Both adequate and inadequate groups had a median flow rate of 3 mL/s, while both groups had a median contrast volume of 80 mL, with no significant differences (*p* = 0.94 and 0.55, respectively) (Table 2). One hundred and twenty-four studies were performed at < 4 mL/s compared to six studies at ≥ 4 mL/s (Table 1). The number of inadequate contrast opacified studies between flow rates of ≥ 4 mL/s and < 4 mL/s was not significantly different (*p* = 0.59) (Table 1).

**TABLE 2:** Technical factors: Flow rate and contrast volume.

Technical factors	Frequency	Mean	Std Dev	Median	Lower Quartile	Upper quartile	Minimum	Maximum	*p*-value
**Flow rate (mL/s)**									0.94
Adequate	103	3.16	0.31	3	3	3.3	2	4	
Inadequate	27	3.12	0.32	3	3	3.5	2	3.5	
**Contrast volume (mL/s)**									0.55
Adequate	103	85.87	14.69	80	80	100	40	110	
Inadequate	27	84.37	16.69	80	70	100	53	120	

Std Dev, standard deviation.

The majority of the CTPAs were performed during the day (73/130, 56.2%). Additionally, the time of the scan acquisition, that is, day versus afterhour shifts and presence of contrast leakage made no significant difference to the rate of inadequate contrast enhancement (*p* = 0.21 and 0.60, respectively) (Table 1).

## Discussion

This audit established that the rate of inadequate CTPAs was higher than the international guideline set by the RCR^7^ and was also greater than international audits (1.5% – 18%).^8,9,10,11^ The inadequate rate of 20.8% in the prospective audit compared closely to the 19.0% in the retrospective audit, which indicates that there was no radiographer bias in the prospective studies. This high rate of inadequate contrast enhancement is undesirable as it contributes to indeterminate studies and can lead to misdiagnosis, repeated examinations, increased radiation dose or unnecessary anticoagulation, all of which are detrimental to the patient.

The effect of the variables (position of IV cannula, size of IV cannula, flow rate, volume of contrast, time of study and presence of contrast leakage) on the rate of inadequate contrast enhancement was found to be non-significant. These findings provide some insights into what does not contribute to inadequate contrast opacification but have to be interpreted in light of the limitations subsequently discussed.

There were higher rates of inadequate contrast enhancement when IV cannulas were positioned in the hand (28.6%), compared to 21.6% in the antecubital fossa and 15.9% in the forearm; however, this small difference was not significant (*p* = 0.65). This is comparable to findings by Marshall et al.^12^ (with a larger sample size of 1500) and Roggenland et al.^13^ These two studies also found no significant difference between different IV cannula sizes, although, similar to our audit, they also had a limited frequency of 22 G IV cannulas. The RCR and American College of Radiology recommends a 20 G or a bigger IV cannula in the antecubital fossa or forearm, but there is no other literature supporting this practice for CTPAs.^7,12,14^ They also do not stipulate recommendations for contrast volume. Contrast opacification is related to iodine flow rate, which is a function of iodine concentration and flow rate.^15^ Our protocol utilised a concentration of 350 mg iodine/mL, which has been shown to be superior to lower concentrations.^16,17^ It can, therefore, be postulated that IV cannula size should only impact contrast opacification when it restricts flow rate. The recommended minimum flow rate for general CT angiograms is 3 mL/s,^14^ while a flow rate of 4 mL/s or higher has been recommended for CTPAs internationally.^13,18,19,20^ Flow rates of 3 mL/s have been safely achieved with 22 G IV cannulae;^21^ thus the use of 22 G cannulas can, in theory, still be sufficient. Power injectors have a predetermined pressure limit that can be exceeded in cases of access vein size and IV cannula size mismatch, and this will lead to an automatic decrease in the flow rate.^22^ Therefore, the IV cannula size and access vein size should be considered as a continuum.

This study only had six CTPAs with a flow rate of ≥ 4 mL/s, and although none of them had inadequate contrast opacification, we could not prove any significant difference in the rate of inadequately contrast-enhanced CTPAs between this and flow rates < 4 mL/s (*p* = 0.59). The significance is limited by the small number of CTPAs with flow rates ≥ 4 mL/s. Several international studies have achieved lower rates of inadequate contrast opacification, compared to ours, utilising flow rates higher than 4 mL/s. Lloyd et al.^11^ achieved a 9% rate of inadequate contrast opacification (< 210 HU), utilising a flow rate of 4 mL/s, while Menon et al.^23^ had < 2% inadequately opacified studies when using a flow rate of 5 mL/s. Similarly, Hendriks et al.^24^ did not have inadequate CTPAs with a flow rate of ≥ 4.2 mL/s; however, they used a less strict cut-off of 180 HU. Ozawa et al.^25^ achieved a significant decrease in inadequate contrast enhancement by increasing flow rate from 2 mL/s to 3 mL/s. Therefore, the high frequency of relatively low flow rates (< 4 mL/s) in this audit might be contributing to the high rate of inadequately opacified studies; however, this requires further investigation. Employing a saline chaser bolus may be helpful in addition to using a higher flow rate.

The contrast volume did not have any significant effect on the rate of inadequate contrast opacification. This is similar to the finding by Goble et al.^17^ who found no significant difference in rates of inadequate contrast opacification between 100 mL and 75 mL (350 mg/mL) contrast volume when using a cut-off value of 250 HU. Another study by Chen et al.^16^ found no significant difference in the rate of inadequately opacified studies between 75 mL and 60 mL (350 mg/mL) contrast volume, also using 250 HU as the cut-off. Individualised contrast volume protocols according to body weight can achieve a reduction in contrast volume while increasing pulmonary arterial enhancement.^24,26^

Other possible explanations for the high rate of inadequately opacified studies can include factors that were not evaluated in this audit such as cardiovascular status, body habitus, patient age, respiratory rate and IV cannula size to access site mismatch. Scanning at maximal inspiration, as utilised in our protocol, has shown inferior contrast enhancement compared to minimal inspiration and expiration.^27,28,29^ Causes for this include transient interruption of contrast and dilution as inspiration causes negative intrathoracic pressure and increased venous return with unopacified blood entering the right heart. It has also been proposed that breath-holding after inspiration leads to a Valsalva effect, with subsequent diminished cardiac output and delayed pulmonary arterial contrast enhancement.^27,30^

A circulation adjusted protocol with scan initiation of 10 s after bolus triggering at 150 HU or a fixed scan delay of 19 s after injection was suggested as adequate.^31^ The use of a multiphasic injection with an exponential decrease in injection rate as opposed to a uniphasic injection rate with a rapid decline results in more uniform and steady-state enhancement, which provides more room for error with regard to timing of scan acquisition.^17^ These may be possible strategies to improve our CTPA scanning protocol which requires further investigation.

### Limitations of the study

The small sample size limited the significance, especially the low number of CTPAs with high flow rates (≥ 4 mL/s) and a single CTPA with a 22 G IV cannula. As this was an audit, variables could not be controlled, which led to an unequal distribution within variables. This audit did not account for patient age, weight, cardiovascular status and respiratory motion, which have all been shown to affect contrast enhancement.^4,13^

The use of one reader limits reliability; however, the HU measurement in the MPA is an objective measurement that requires minimal expertise and the addition of the retrospective audit improved the reliability. The amount of contrast leakage was not quantified.

### Recommendations

The CTPA protocol at this hospital should be assessed and adjusted to achieve a lower rate of inadequate contrast enhancement. This will lead to less indeterminate studies, less repeated studies and improved patient care. Alterations that have been advised and shown to be helpful include scanning at minimal inspiration or expiration,^27,28,29^ changing timing after bolus tracking, use of a saline chaser and use of a test bolus.^7^ Although this study found no significant difference in the effect of IV cannula size on the rate of inadequately opacified CTPAs, we do not have sufficient evidence to support the use of 22 G cannulas; therefore, the use of a 20 G or larger cannula is still preferred and is in line with current international recommendations. We suggest that CTPAs not be cancelled when IV access is peripheral to the antecubital fossa if it can tolerate acceptable flow rates, as we did not find any significant difference in the rate of inadequately opacified studies with IV access distal to the antecubital fossa. We do not have enough evidence for an adequate minimum flow rate, but the limited findings of this audit together with international findings in favour of high flow rates (≥ 4 mL/s) warrant the investigation of higher flow rates in our setting. Further studies are still required to assess the minimum required flow rate for consistent adequate contrast opacification.

The use of protocols adjusting contrast volume according to body weight and using a multiphasic injection can be investigated. Following any changes to current practice, further audits should be performed to assess any subsequent improvement in the rate of inadequate opacification.

We recommend that similar audits be performed at other institutions to ensure that adequate contrast enhancement is achieved and local guidelines are established.

## Conclusion

This audit proves that the rate of inadequately enhanced CTPAs at this hospital, according to the RCR recommendations, was high. Causes for inadequate contrast enhancement are multifactorial, and we could not prove any significant impact with regard to the size of IV cannula, position of IV cannula, volume of contrast, time of study acquisition and presence of contrast leakage on the rate of inadequately opacified CTPAs. Although evidence from this study is limited, the use of high flow rates (≥ 4 mL/s) should be explored in an attempt to reduce inadequate opacification.
